# 3-Methoxy Carbazole Impedes the Growth of Human Breast Cancer Cells by Suppressing NF-κB Signaling Pathway

**DOI:** 10.3390/ph15111410

**Published:** 2022-11-14

**Authors:** Jowaher Alanazi, Aziz Unnisa, Muteb Alanazi, Tareq Nafea Alharby, Afrasim Moin, Syed Mohd Danish Rizvi, Talib Hussain, Amir Mahgoub Awadelkareem, AbdElmoneim O. Elkhalifa, Syed Shah Mohammed Faiyaz, Mohammad Khalid, Devegowda Vishakante Gowda

**Affiliations:** 1 Department of Pharmacology and Toxicology, College of Pharmacy, University of Ha’il, Ha’il 81442, Saudi Arabia; 2 Department of Pharmaceutical Chemistry, College of Pharmacy, University of Ha’il, Ha’il 81442, Saudi Arabia; 3 Department of Clinical Pharmacy, College of Pharmacy, University of Ha’il, Ha’il 81442, Saudi Arabia; 4 Department of Pharmaceutics, College of Pharmacy, University of Ha’il, Ha’il 81442, Saudi Arabia; 5 Department of Clinical Nutrition, College of Applied Medical Sciences, University of Hail, Ha’il 81442, Saudi Arabia; 6 Department of Physiology, College of Medicine, University of Ha’il, Ha’il 81442, Saudi Arabia; 7 Department of Pharmacognosy, College of Pharmacy, Prince Sattam Bin Abdilaziz, Al-Kharj 11942, Saudi Arabia; 8 Department of Pharmaceutics, Cauvery College of Pharmacy, Mysuru 570028, India

**Keywords:** 3-Methoxy carbazole, natural compounds, breast cancer, anti-cancer, MCF-7 cells

## Abstract

Breast cancer represents the most frequently occurring cancer globally among women. As per the recent report of the World Health Organization (WHO), it was documented that by the end of the year 2020, approximately 7.8 million females were positively diagnosed with breast cancer and in 2020 alone, 685,000 casualties were documented due to breast cancer. The use of standard chemotherapeutics includes the frontline treatment option for patients; however, the concomitant side effects represent a major obstacle for their usage. Carbazole alkaloids are one such group of naturally-occurring bioactive compounds belonging to the Rutaceae family. Among the various carbazole alkaloids, 3-Methoxy carbazole or C_13_H_11_NO (MHC) is obtained from *Clausena heptaphylla* as well as from *Clausena indica*. In this study, MHC was investigated for its anti-breast cancer activity based on molecular interactions with specific proteins related to breast cancer, where the MHC had predicted binding affinities for NF-κB with −8.3 kcal/mol. Furthermore, to evaluate the biological activity of MHC, we studied its in vitro cytotoxic effects on MCF-7 cells. This alkaloid showed significant inhibitory effects and induced apoptosis, as evidenced by enhanced caspase activities and the cellular generation of ROS. It was observed that a treatment with MHC inhibited the gene expression of NF-kB in MCF-7 breast cancer cells. These results suggest that MHC could be a promising medical plant for breast cancer treatment. Further studies are needed to understand the molecular mechanisms behind the anticancer action of MHC.

## 1. Introduction

Among the different carcinomas reported globally, breast cancer represents the most common malignancy with a low survival rate, globally, among women. As per the recent report of the World Health Organization (WHO), it was documented that by the end of year 2020, approximately 7.8 million females were positively diagnosed with breast cancer and in 2020 alone, 685,000 casualties were documented due to breast cancer [1]. From the perspective of cancer biology, breast cancer is a highly heterogenous malignancy and it is further subdivided on the basis of an expression of receptors and/or molecular heterogeneity into different subtypes. Among its different subtypes, triple negative breast cancer, or TNBC, itself constitutes approximately 25% of all the reported incidences of breast cancer. TNBC is primarily characterized as tumors that exhibit a negative expression of estrogen, prostate and/or receptors and human epidermal growth factor [2,3]. Another peculiar attribute of TNBC among the other subtypes of breast cancer is its invasive and highly metastatic behavior which is extremely progressive. Furthermore, the patients diagnosed with TNBC are commonly younger females, i.e., pre-menopausal females, and relatedly, there is also an increased possibility for disease recurrence, and a lower survival rate [3,4,5,6].

Nuclear factor kappa B, or NF-κB, recognized during late 20th century, is an important transcription factor which is implicated in several debilitating human diseases of which inflammatory, viral, and metabolic disorders are noteworthy. Researchers have also previously associated the expression of NF-κB with unregulated cellular proliferation and oxidative imbalance [7,8,9]. Intriguingly, several reports have previously indicated that a chronic expression and activation of NF-κB is associated with an augmented progression of different cancers including that of the colon, breast, rectum, lung and stomach [10,11]. The chronic expression of NF-κB further modulates the expression of different genes, namely, B-cell lymphoma-2 (Bcl-2), which mediates apoptotic cell death within tumor cells, and caspase-3, which is actively involved in regulating the apoptotic cell death [12,13]. Other genes that are influenced by NF-κB expression include cyclin D1, cyclooxygenase-2, vascular endothelial growth factor (VEGF), and interleukin 8 (IL-8), along with the genes favoring an epithelial–mesenchymal (EMT) transition [14,15,16,17]. Previously it has been reported that impeding the expression of NF-κB could result in the instigation of apoptotic cell death in different cancer cells with a concomitant reduction in the invasive potential and proliferation of cancer cells, resulting in an enhanced sensitivity towards chemotherapeutics [18,19,20]. Thus, it can be concluded that NF-κB can serve as an important therapeutical target for the treatment of cancer including the dreaded breast cancer. 

It has now been established that various naturally-active, bioactive compounds possess a great diversity in both their structure and their mode of action in the clinical management of cancer. Presently, around 60% of all the cancer chemotherapeutics being used for the clinical management of cancer are natural products of their isolates. This evidence thereby indicates the relevance of natural compounds for their further exploration in formulating novel chemotherapeutics against cancer [21,22]. Carbazole alkaloids are one such group of naturally-occurring bioactive compounds belonging to the Rutaceae family. Previously, these have been reported to possess anti-cancer attributes by intercalating DNA, impeding DNA replication and exerting antagonistic effects on estrogen receptors [23,24]. Intriguingly, different carbazole-based agents are already approved and are being used for the clinical management of different cancers [25], explicitly demonstrating their abilities for being prerequisite anticancer therapeutical candidates. Common phyto-carbazole alkaloids occurring in nature include koenimbine, koenigicine, mahanimbine and clausazoline-K that possess anti-lipase properties, whereas pyrayafoline-D, murrafoline-I, mahanimbine and mahanine have been documented to exert anticancer effects via the instigation of apoptotic cell death by activating the caspase pathways [26]. In the present article, we have deciphered the anticancer and apoptotic potential of 3-Methoxy carbazole (MHC) against breast cancer cells via an in silico and in vitro approach. Although various carbazole alkaloids have been explored, the efficacy of MHC has not yet been studied.

## 2. Results

### 2.1. Molecular Docking Results

**Figure 1:**
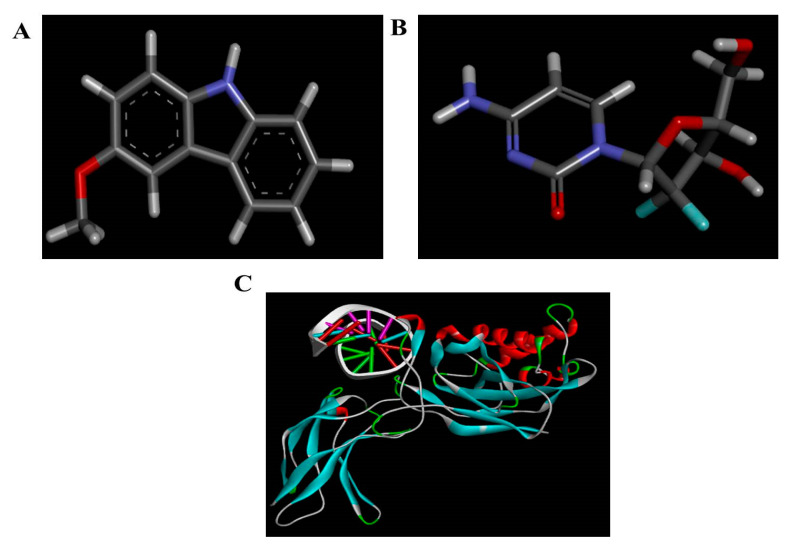
**Three-dimensional** chemical structures of (**A**) 3-Methoxy carbazole, (**B**) Gemcitabine and (**C**) NF-κB.

Three-dimensional structures of 3-Methoxy carbazole (MHC) were downloaded from the PubChem database. These compounds were docked to the NF-κB precursor protein p105, to predict the mechanism of breast cancer cell suppression. In this study, the commercial anticancer drug, gemcitabine, was selected as a reference compound (Figure 1A–C).

### 2.2. Interaction and Binding Affinity between Carbazole Alkaloids and NF-κB

**Figure 2:**
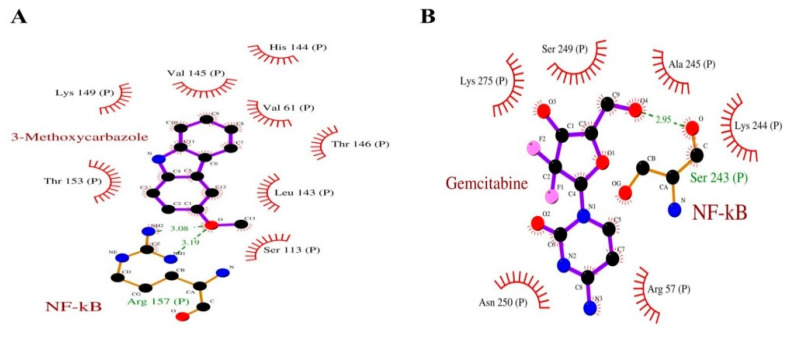
Ligplots showing the molecular interaction of the NF-κB precursor p105 with (**A**) 3-Methoxy carbazole and (**B**) Gemcitabine.

Our docking studies predicted NF-κB binding energies of MHC higher than −5.8 kcal/mol, which is the binding energy of gemcitabine (Table 1). The residues involved in the hydrophobic interaction with gemcitabine were Ala245, Lys244, Arg57, Asn250, Lys275, Ser249, and Ser243, that engaged in hydrogen bonding (with bond lengths of 2.95). The MHC was predicted to interact with the NF-κB by amino acids involved in the hydrogen bonding and hydrophobic interactions such as Leu143, Ser113, Thr153, Lys149, Val145, His144, Val61, and Thr146; however, Arg157 was engaged in the hydrogen bond interactions (with bond lengths of 3.19 and 3.08) and the binding energy was found to be −6.0 kcal/mol (Figure 2A,B). 

### 2.3. MHC Decreased MCF-7 Cells Viability 

**Figure 3:**
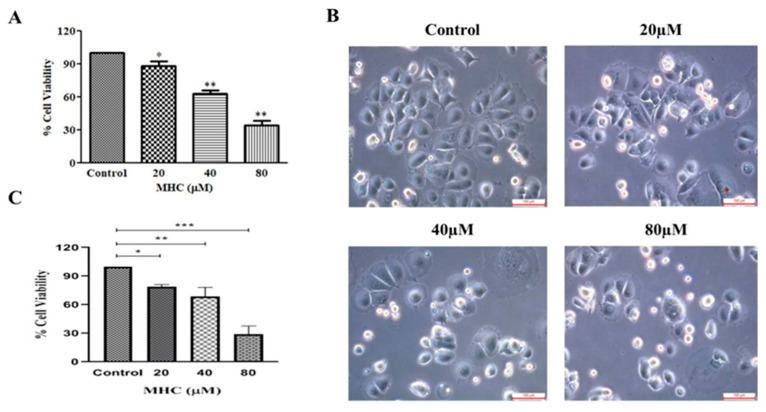
Anti-cancer effect of MHC against MCF-7 cells: (**A**) cellular viability post-MHC treatment after 24 h, (**B**) photomicrographs indicating the morphological alterations and (**C**) cell viability % of MDA-MB-231 in response to MHC exposure. Scale bar 100 µm. * *p* < 0.05, ** *p* < 0.01 and *** *p* < 0.001.

To investigate the plausible cytotoxicity of MHC on MCF-7 cells, an MTT assay was performed after treating MCF-7 cells with different concentrations of 20, 40 and 80 µM for 24 h. The results indicated that the MHC significantly impeded the growth of the MCF-7 cells by 88.40 ± 3.50% (20 µM; *p* < 0.05), 62.88 ± 2.77% (40 µM; *p* < 0.01), and 34.08 ± 3.73% (80 µM; *p* < 0.001), in a dose-dependent manner (Figure 3A). Furthermore, a morphological analysis of the MCF-7 cells upon the treatment with MHC was evaluated through phase-contrast microscopy. As illustrated in Figure 3B, the photomicrographs positively indicated towards the shrinkage, swelling and rupturing of the MCF-7 cells; thereby, confirming the dose-dependent cytotoxic effect of MHC on breast cancer cells. Furthermore, the MHC exhibited its competence in exerting substantial cytotoxic effects on MDA-MB-231 cells. The observations from the MTT assay elucidated that the MHC restricted the growth of the MDA-MB-231 cells by 85.33 ± 4.02 (*p* < 0.05), 68.71 ± 4.46 (*p* < 0.01) and 32.19 ± 3.69 (*p* < 0.001) at concentrations of 20 µM, 40 µM and 80 µM, respectively (Figure 3C). 

**Figure 4:**
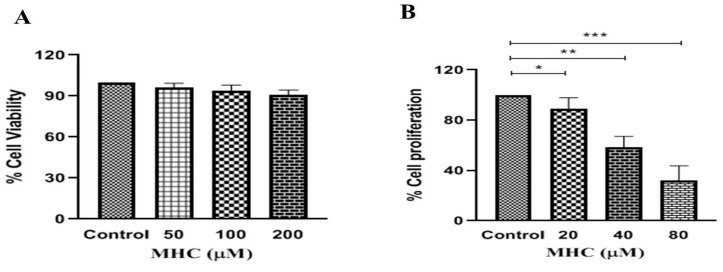
(**A**) MHC-mediated non-cytotoxic effects against human-lung WI38 cells post-MHC treatment after 24 h and (**B**) the effect of MHC in impeding the proliferation of MCF-7 cells as per the observations of the BrdU assay. * *p* < 0.05, ** *p* < 0.01 and *** *p* < 0.001.

Intriguingly, as shown in Figure 4A, the MHC failed to exert any cytotoxic effects on human-lung WI38 non-cancerous cells. The viability of the WI38 cells post-MHC exposure at varying concentrations was found to be >90%. 

### 2.4. MHC Impeded Proliferation of MCF-7 Cells

The anti-proliferative effects of the MHC at concentrations of 20, 40 and 80 µM on the MCF-7 cells were investigated using a BrdU assay. The observations from this investigation explicitly showed that the MHC was competent in reducing the proliferation of MCF-7 cells by 89.33% ± 4.02% at a concentration of 20 µM (*p* < 0.05). The results also elaborated that the MHC exhibited a dose-dependent effect in reducing the proliferation of MCF-7 cells by 58.38% ± 4.12% and 32.19% ± 4.54% at 40 µM and 80µM concentrations (*p* < 0.01 and *p* < 0.001) respectively, (Figure 4B). 

### 2.5. MHC-Induced Apoptosis 

**Figure 5:**
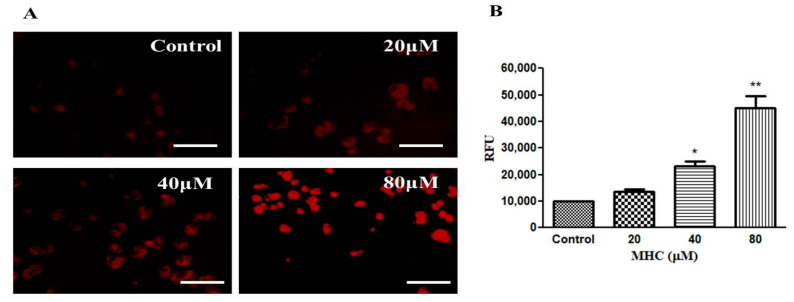
MHC-mediated instigation of apoptosis within MCF-7 cells as evaluated through (**A**) fluorescent photomicrographs of MCF-7 cells stained using PI and (**B**) its quantification. Scale bar 100 µm. * *p* < 0.05, and ** *p* < 0.01.

MHC-induced apoptosis within the MCF-7 cells was quantified using PI staining followed by counting the number of cells going through apoptosis via fluorescence microscopy. As observed in the micrographs of Figure 5A, the apoptotic cells were characterized by an increased red fluorescence indicating the formation of apoptotic bodies in the MHC-treated MCF-7 cells. Moreover, untreated control cells were distinguished by the presence of a diffuse red fluorescence due to the presence of intact nuclei within the cells. At the highest dose (i.e., 80 µM) of MHC, nuclear chromatin condensation and blebbing were observed, which are considered to be peculiar attributes of apoptosis. The results showed MHC-induced morphological aberrations in the nucleus following a dose-dependent trend, which ultimately lead to apoptosis. Moreover, the MHC-induced apoptosis was quantified in the MCF-7 cells. As shown in Figure 5B, a dose-related increase in apoptotic cells with a bright red fluorescence was observed as compared to the control. Therefore, the stated observations indicated that the MHC exposure considerably instigated apoptosis within the MCF-7 cells. 

### 2.6. MHC Elevated Caspase-3 Activity

**Figure 6:**
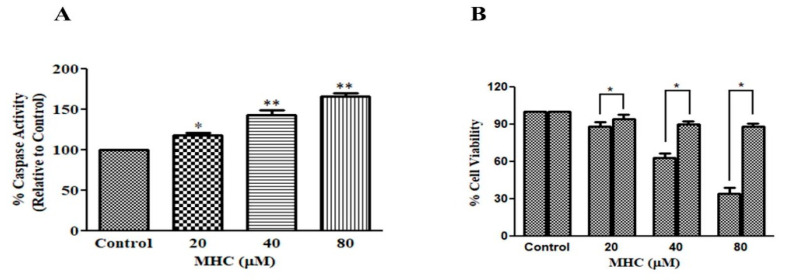
(**A**) Alteration in the levels of caspase-3 post treatment with MHC and (**B**) cell viability of MCF-7 cells treated with a caspase-3 inhibitor. * *p* < 0.05 and ** *p* < 0.01.

Caspase-3 is an important arbitration of the caspase signaling cascade resulting in the instigation of apoptosis. The investigators tried to substantiate whether MHC-instigated apoptosis altered the expression of capase-3 comparatively with untreated, control MCF-7 cells. The observations were clearly indicative of the notion that an MHC exposure succeeded in elevating the capsase-3 activity within A549 cells. The respective activity was elevated by 18.40 ± 2.50% (20 µM), 43.21 ± 3.99% (40 µM), and 66.42 ± 3.19% (80 µM) within the MCF-7 cells (Figure 6A).

### 2.7. Attenuation of MHC-Induced Apoptosis by Caspase-3 Inhibitor

To characterize whether MHC-induced cytotoxicity in breast cancer MCF-7 cells was due to the activation of caspase-3, MCF-7 cells pre-exposed to Z-DEVD-FMK were evaluated for MHC-mediated cytotoxicity. The pretreatment with the capase-3 inhibitor substantially impeded the MHC-mediated cytotoxicity within the MCF-7 cells (Figure 6B). Such an observation was indicative of the notion that the activation of caspase-3 was important for the MHC-induced apoptosis.

### 2.8. Evaluation of MHC Effects on ROS Alteration within MCF-7 Cells

**Figure 7:**
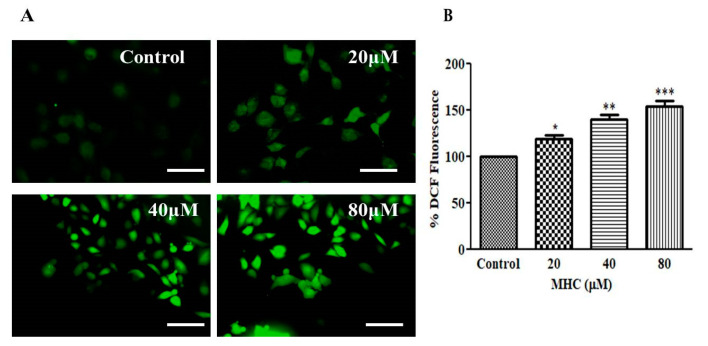
MHC-mediated instigation of intracellular ROS as assessed through (**A**) DCF-DA stained fluorescent photomicrographs and (**B**) quantification of DCF-DA-mediated mean fluorescence intensity within MCF-7 cells. * *p* < 0.05, ** *p* < 0.01 and *** *p* < 0.001.

An increased level of ROS production is positively associated with the initiation of apoptotic pathways within cancerous cells [27]. Thus, the investigators believed that a determination of the ROS levels within MCF-7 breast cancer cells was important to measure post-MHC dosing at the stated concentrations. As shown in Figure 7A,B, a substantial increase of the ROS level by 19.38 ± 3.42% was seen as compared to the untreated cells, following treatment with the 20 µM of MHC. Intriguingly, the ROS generation was further enhanced to 39.68 ± 4.19% and 53.75 ± 5.05% in the MCF-7 cells at the concentrations of 40 µM and 80 µM, respectively. The stated observations were indicative of the notion that the MHC treatment augmented the ROS production in the breast cancer MCF-7 cells.

### 2.9. MHC Treatment deflated NF-κB Levels

**Figure 8:**
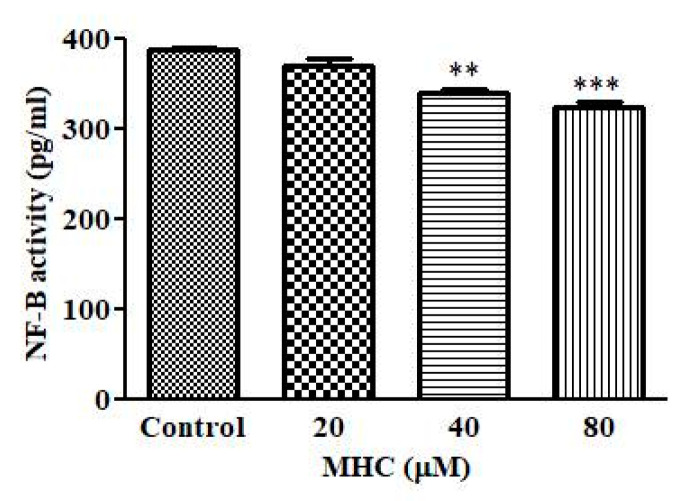
Alterations in the levels of NF-κB post-exposure with MHC. ** *p* < 0.01 and *** *p* < 0.001.

It is now an established fact that NF-κB is pivotal in the survival and proliferation of cancer cells. Therefore, the effect of MHC in regulating NF-κB levels was also assessed using a sandwich ELISA. The results as shown in Figure 8 indicated that no substantial effect on the NF-κB levels was seen at a 20 µM concentration; however, the level of NF-κB was significantly reduced at 40 µM (*p* < 0.01) and 80 µM (*p* < 0.001) concentrations of MHC.

## 3. Discussion

The constitutive activation and/or expression of the NF-κB pathway is responsible for positively regulating cell proliferation, and tumor growth with a concomitant suppression of apoptotic cell death during breast cancer [27]. Elevated NF-κB DNA-binding activity has been established in pre-clinical breast cancer models. Intriguingly, p50/RelA heterodimer was documented to be related in nearly 86% of estrogen receptor (ER)-negative and Her2/ErbB2-positive breast tumors [28]. Indeed, it has been previously reported that an activation of NF-κB is induced by Her2/ErbB2 via modulation of the PI3K/Akt pathway where the degradation of IκBα is achieved through calpain [29]. In fact, ERs have been previously reported for negatively regulating NF-κB through different mechanisms [30]. 

Bioactive constituents obtained from natural resources have shown their relevance for being potent anti-cancer therapeutics. Among these bioactive constituents, the derivatives of carbazole alkaloid hold the potential for compromising DNA integrity within cells by inhibiting the functioning of the DNA replication enzymes, which in turn restricts the functioning of different kinases and acts as an antagonist for estrogen receptors. Consequently, we chose MHCs for our present study as they could be useful lead hits/candidates for the discovery of novel anticancer agents. In the present article, we aimed to validate the efficacy of carbazole alkaloids (MHCs) through molecular docking and to delineate the mechanistic action of these compounds against p105, a transcription factor governing the NF-kB over-expression in breast cancer. We also investigated the cytotoxic potential of MHC on MCF-7 breast cancer cells. Our docking results illustrated that carbazole alkaloids could act as plausible NF-κB inhibitors since they preliminarily exhibited their binding with p105 by interacting with the RH domain containing important sequences required for DNA binding and dimerization. It was observed that the best efficacy of binding with the NF-κB RH domain was elucidated by the MHC. Since these alkaloids bind p105 in their RH domain, there is the possibility of only two outcomes. Firstly, either the resulting p50 dimerization could be impeded or secondly, as in the case where the p50 succeeded in forming a functional homo- and/or hetero-dimer with other Rel/NF-κB family members, the DNA binding of these functional dimers at the kB sites would be hindered, and ultimately, the genes which were regulated by NF-κB would not be expressed. Thus, the MHC exhibited its potential for being a plausible inhibitor of the NF-κB pathway. These observations led the investigators to hypothesize that MHC, by interacting with the NF-κB DNA binding or RH domain, could be the rationale behind any impeded NF-κB activities. 

Furthermore, we substantiated the anti-cancerous effects of the MHC against breast cancer MCF-7 and MDA-MB-231 cell lines. The analysis of the MCF-7 and MDA-MB-231 cell viability by an MTT assay suggested that MHC strongly reduced the growth in these cell lines. Importantly, during this investigation it was also found that MGC was safe towards normal cells, since it failed to instigate any considerable cytotoxic effects against non-cancerous human WI38 cells. Furthermore, a morphological analysis by a phase contrast microscopy showed that the morphological features of the cells were changed significantly, post-MHC-exposure and that they were represented by cellular shrinkage and cluster-forming cells that were detached previously from the surface; therefore, indicating the therapeutical relevance of MHC in a dose-dependent manner for the management of breast cancer cells. The BrdU-mediated cell proliferation assay further corroborated that the MHC exerted restraining effects on the proliferation of the MCF-7 cells which could plausibly be attributed to its modulatory effect on genes associated with the cell cycle progression. 

Preceding reports have indicated that most of the chemotherapeutic agents reduce cancer cell proliferation by inducing apoptosis. Apoptotic cell death is peculiarly characterized by condensation with chromatin and a fragmentation of the nuclei followed by a circular cell morphology, reduced cell volume and formation of apoptotic bodies [31]. Our results also support the above notion, where the apoptotic nuclei in the MCF-7 cells suggested that the MHC induced apoptosis in breast cancer cells. Synthesized caspases are in the form of inactivated proenzymes whose activation during apoptosis is mediated by cleavage at particular regions containing aspartate. Caspase-3 has previously been reported as an important mediator of apoptotic cell death by the proteolytic degradation of proteins [32]; therefore, the activity of caspase-3 was investigated and it was inferred that the MHC induced the caspase-3 activation, leading to apoptosis. Pretreatment with a specific inhibitor impeded the MHC-instigated toxicity in the MCF-7 cells, indicating caspase-3 activation during the MHC-induced apoptosis. It is now an established notion that an NF-κB over-activation is related positively with several human cancers [33]. Moreover, it also plays an important role in regulating the transcriptional activation of anti-apoptotic genes [34]. Thus, it is believed that reducing the NF-κB functionality can be considered an impetus for triggering apoptosis. Here, it was established that MHC was competent in lowering NF-κB levels in MCF-7 cells; therefore, it was deduced that the MHC-mediated instigation of apoptosis in MCF-7 cells could plausibly be correlated with a suppressed NF-κB activity. Conclusively, MHC could be a plausible therapeutic modality in the treatment of breast cancer. In view of the above discussion, the investigators believe that although MHC could serve to be a potent chemotherapeutic for the management of triple negative breast cancer, before reaching to such a conclusion, further studies are warranted. Therefore, an exhaustive molecular elucidation of MHC-mediated effects on established, preclinical in vivo models of breast cancer could be the next research interest in this field.

## 4. Materials and Methods

### 4.1. Materials

The 3-Methoxy carbazole (MHC) was purchased from Sigma Aldrich, St. Louis, MO, USA. The 3-(4,5-dimethylthiazol-2-yl)-2,5-diphenyl-2H-tetrazolium bromide (MTT) dye was commercially obtained from Himedia, India. 2′,7′-Dichlorofluorescin diacetate (DCF-DA), propidium iodide (PI) and Z-DEVD-FMK, a caspase-3 inhibitor, were procured from Sigma. Eagle’s minimum essential medium (MEM) with Earle’s salt, fetal bovine serum (FBS), and antibiotic–antimycotic solution were commercially obtained from Gibco. The caspase-3 colorimetric assay kit that was used was purchased from BioVision and the human NF-κB (p105/p50) ELISA Kit (ab278120) was purchased from Abcam.

### 4.2. Methods

#### 4.2.1. In Silico Investigations

##### Retrieval of Protein 3D Structure

The crystal structure of the NF-κB (PDB: 1SVC) taken in this study was extracted from the Brookhaven Protein Data Bank (http://www.rcsb.org/pdb) accessed on 20 August 2022. The structure of the NF-κB used for docking was devoid of all the heteroatoms including non-receptor atoms such as ions, water, etc. 

##### Retrieval of Ligands 3D Structure

The required ligands were searched for on the database of PubChem (http://pubchem.ncbi.nlm.nih.gov) accessed on 20 August 2022. The structural and functional details about the different organic compounds were retrieved from the database of PubChem having a unique CID or compound identification number. The structural details about the desired ligands were collected using a Simplified Molecular Input Line Entry Specification string, which was further deposited in the CORINA (http://www.molecular-networks.com/prod-ucts/corina) accessed on 20 August 2022 software. This software utilizes the SMILES string to create a 3D structure of the desired molecules which can then be retrieved in PDB format for performing an AutoDock Vina 4.0 [35]. Based on the previously available material, the binding pocket coordinates were determined, and the grid box was input within a cubic box of magnitudes 40 × 40 × 40 [36]. 

##### Visualization of Docked Complex

The best-docked position was selected from among nine possible configurations based on the interacting residues, such as hydrogen bonds with a high binding affinity (kcal/mol). The protein–ligand interaction of the docked complexes was visualized in two dimensions using LigPlot [37], and PyMol was used to generate all the binding pockets [38]. Visualization of the protein–ligand docked complex was performed using LigPlot (http://biochem.ucl.ac.uk/bsm/ligplot/ligplot.html) accessed on 20 August 2022, and was further aided by a ghost script viewer. LigPlot represents a command line-based program commonly employed for acquiring the automated plots of interactions occurring between protein–ligand interactions from 3D protein−ligand interaction-based coordinates, and this results in the generation of schematic illustrations depicting the residues of proteins and ligands involved in an interaction [38]. These interactions are mediated by hydrogen bonds or may arise due to hydrophobic interactions. The representation for the hydrogen bonds includes dashed lines in between the interacting atoms, whereas the regions of the hydrophobic interactions are depicted by an arc with spokes radiating towards the atoms within the ligand interacting within the protein. The atoms contacted are represented with spokes radiating backwards. 

#### 4.2.2. In Vitro Validation 

##### Cell Line and Culture Maintenance

Human, estrogen-positive breast cancer-derived MCF-7 and MDA-MB-231 cells, along with human-lung WI-38 non-cancerous cells were obtained from the cell repository of the National Center of Cell Sciences, Pune, India. The same cells were cultured in Eagle’s minimum essential medium (MEM) with Earle’s salt. The media was supplemented with 10% fetal bovine serum (FBS) and a 1% anti-biotic and anti-mycotic solution. The culture was continuously maintained under an ambient tissue culture environment comprising of 5% CO_2_ at 37 °C. 

##### Cytotoxicity Evaluation 

The cytotoxic effects of MHC against the human breast cancer MCF-7 and MDA-MB-231 cells was quantified using tetrazolium MTT dye as previously described [39]. Briefly, 1 × 10^4^ MCF-7 and MDA-MB-231 cells were exposed to various doses of MHC for 24 h and subsequently, the media with the dissolved MHC was replaced with the MTT dye (10 µL; 5 mg/mL), then, the plate was again incubated for 4 h. Thereafter, each well was supplemented with 100 µL of tissue culture grade DMSO and the reaction was provided a brief incubation of 30 min in the dark at 37 °C. Finally, the absorbance of dissolved formazan was quantified by recording the absorbance at 570 nm through a spectrophotometer (Bio-Rad, Hercules, CA, USA). The cytotoxicity of the MHC against the MCF-7 cells was interpolated in terms of the cell viability percentage (%) by using the formula:

Cellular viability % = (Absorbance of treated MCF-7 cells)/(Absorbance of untreated MCF-7 control cells) × 100

The cytotoxicity of the MHC against the normal human-lung WI38 cells was evaluated using the same procedure.

##### Assessment of Cell Proliferation

The MHC-mediated effect on the proliferation of MCF-7 cells was estimated through a BrdU assay colorimetrically using an ELSIA kit (Roche, Basel, Switzerland) as per the protocol reported previously [40]. Concisely, the BrdU incorporates itself in exchange with thymidine within the DNA of the dividing cells. BrdU is subsequently identified by peroxidase-conjugated anti-BrdU antibodies. The end product of this reaction was eventually quantified by recording its absorbance at 370 and 492 nm as reported previously [41,42] using a microplate reader (Bio-Rad, Hercules, CA, USA). The interpretations of the results were expressed in terms of the percentage (%) of cellular proliferation in comparison with the untreated control using the formula:

% Cell proliferation = [(Absorbance_TREATED_ at 370 nm − 492 nm)/Absorbance_CONTROL_ at 370 nm − 492 nm)] × 100

##### Assessment of Intracellular Reactive Oxygen Species Generation

The intracellular estimation of the ROS was quantified using a DCF-DA stain through a spectrophotometer as reported previously [43]. A total of 1 × 10^4^ MCF-7 cells were incubated overnight for adherence in each well of a 96-well plate under the optimum culture conditions. The cells were then subjected to 20, 40 and 80 µM of MHC for 12 h. Subsequently, the cells were exposed to 10 mM of DCFH-DA at 37 °C for additional 30 min in the dark. Finally, the DCF-DA-mediated fluorescence intensity (excitation:emission = 458/528 nm) was quantified using a microplate reader (BioTek, Winooski, VT, USA). 

##### Analysis of Apoptosis

For assessing the efficacy of MHC in instigating apoptosis within MCF-7 cells, the PI staining and fluorescence was quantified using the ImageJ software (NIH, Bethesda, MA, USA). Nearly 5 × 10^3^ cells/well were allowed to adhere overnight under standard culture conditions. Subsequently, the cells were exposed to varying concentrations (20, 40 and 80 µM) of MHC and the plates incubated for 24 h. The fluorescence of the PI stain was recorded using red filters at an excitation:emission wavelength of 586/15 nm:646/68 nm of a FLoid Imaging station (Thermo-Scientific, Waltham, MA, USA). Subsequently, the fluorescence was quantified using the ImageJ software (NIH, Bethesda, MA, USA).

##### Analysis of Caspase-3 Activity

The caspase-3 activity was assayed using a Caspase-3 Colorimetric Assay Kit (Bio-Vision, Milpitas, CA, USA). Initially, 3 × 10^6^ cells were incubated with 20, 40 and 80 µM of MHC for 24 h. The treated and/or untreated cells were subjected to lysis using a lysis buffer (50 µL) by briefly (10 min) incubating on ice. The resulting lysate was further subjected to centrifugation (10,000× *g*; 1 min and 4 °C). Subsequently, the lysate was re-diluted using a 50 mL dilution buffer after quantifying the protein levels in the different groups. An amount of 50 µL of the obtained lysate was taken in each well of a 96-well plate and mixed with an equal volume of the reaction buffer (containing DTT; 10 mM) that was added to each sample. Thereafter, a DEVD-pNA substrate (4 mM; 5 µL) was also added in each well and the plate was incubated for 1 h (37 °C). Finally, the plate was read for its absorbance which was recorded at 405 nm using a microtiter plate reader. The alteration within the caspase-3 activity was expressed as a percentage (%) increase by comparing these results with the level of the control.

##### Analysis of the Effect of Caspase-3 Inhibitor 

The MHC-instigated cytotoxicity on the MCF-7 cells was characterized using a caspase-specific inhibitor. The cells were subjected to a pre-treatment (2 h) of the caspase-3 inhibitor, Z-DEVD-FMK (50 mM), and thereafter, the cells were again treated with the above-stated MHC concentrations for 24 h. Finally, the viability percentage of the MCF-7 cells was evaluated through an MTT assay as stated above. 

##### Estimation of the NF-κB Levels

In order to study the effect of MHC on the expression of NF-κB (p105/p50) in human breast cancer MCF-7 cells, the expression of NF-κB was estimated as per the manufacturer’s instructions of a Human NF-kB (p105/p50) ELISA Kit (ab278120).

### 4.3. Statistical Evaluation

The data reported was a mean ± standard error of mean (SEM) of three independent experiments performed thrice. A one-way ANOVA followed by Dunnett’s multiple comparison tests were used for the statistical interpretation of the observed values through the Graph Pad prism software (Ver. 5). The differences in *p* values (* *p* < 0.05, ** *p* < 0.01, and *** *p* < 0.001) were identified as being statistically significant.

## Figures and Tables

**Table 1:** Molecular interaction studies of 3-Methoxy carbazole and Gemcitabine with the NF-κB precursor p105.

Interacting Molecules	Binding Energy (Kcal/mol)	Interacting Residues
NF-κB-Gemcitabine	−5.8	Ala^245^, Lys^244^, Arg^57^, Asn^250^, Lys^275^, Ser^249^, and Ser^243^
NF-κB-Gemcitabine-3-Methoxy carbazole	−6.0	Leu^143^, Ser^113^, Thr^153^, Lys^149^, Val^145^, His^144^, Val^61^, Thr^146^, and Arg^157^

## Data Availability

Data is contained within the article.
